# Giant Recurrent Retroperitoneal and Ischioanal Lymphangioma in an Adult: A Case Report

**DOI:** 10.3390/reports9030312

**Published:** 2026-09-14

**Authors:** Edy Quizhpe, Alex Paúl Guachilema Rivadeneira, Jaime Eduardo Ortíz Flores, Melany Micaela Montalvo Cevallos

**Affiliations:** 1 Facultad de Ciencias de la Salud “Eugenio Espejo”, Universidad UTE, Quito 170527, Ecuador; 2 Hospital General del Sur de Quito, Instituto Ecuatoriano de Seguridad Social (IESS), Quito 170132, Ecuador; 3 Hospital General Enrique Garces, Ministerio de Salud Pública (MSP), Quito 170606, Ecuador; aleix220509@gmail.com (A.P.G.R.); eduormh@gmail.com (J.E.O.F.); 4 Facultad de Salud y Bienestar, Pontificia Universidad Católica del Ecuador, Quito 170525, Ecuador; mel._mic@hotmail.com

**Keywords:** lymphatic malformation, lymphangioma, ischioanal fossa, retroperitoneal, multidisciplinary surgery

## Abstract

**Background and Clinical Significance:** Lymphatic malformations (cystic lymphangiomas) are rare benign tumors in adults, with retroperitoneal and ischioanal involvement being exceptionally uncommon. These lesions often present significant surgical challenges because of their infiltrative growth pattern and high risk of recurrence following incomplete excision. We present a rare case of a giant recurrent retroperitoneal lymphangioma with extension into the pelvic and perineal regions after two previous unsuccessful surgical interventions; **Case Presentation:** A 46-year-old Hispanic female presented with progressive pelvic and perineal swelling associated with discomfort and functional limitation. The patient had a history of two prior surgeries for lymphangioma recurrence. Imaging studies revealed a large multicystic retroperitoneal mass extending through the pelvis into the ischioanal fossa and perineal region. Given the lesion’s size and complex anatomical involvement, a multidisciplinary surgical approach was planned. The patient underwent a combined abdominal–perineal en bloc resection, achieving complete removal of a 20 × 8 × 9.5 cm mass while preserving anal sphincter integrity and function. Histopathological examination confirmed the diagnosis of cystic lymphangioma. The postoperative course was uneventful, and no recurrence was observed for 6 months follow-up; **Conclusions:** Giant recurrent pelvic lymphangiomas require complete en bloc excision and a multidisciplinary approach to ensure disease clearance and prevent recurrence.

## 1. Introduction and Clinical Significance

Lymphatic malformations (LMs), formerly known as lymphangiomas, are benign vascular lesions resulting from abnormal development of the lymphatic system. According to the most recent classification system by the International Society for the Study of Vascular Anomalies (ISSVA), these are categorised as macrocystic, microcystic, or mixed lymphatic malformations [1]. While the condition predominantly affects children, with 90% of diagnoses occurring before the age of two, cases in adults are extremely rare, with a very low estimated incidence of hospital admissions [2,3]. In adults, the cause may be an acquired secondary condition resulting from lymphatic obstruction caused by previous trauma, surgery, inflammation, or radiotherapy [4]. Furthermore, adult-onset LMs are frequently considered dormant congenital remnants that late-proliferate due to local factors, infection, or hormonal changes [5]. When localized strictly within the retroperitoneal space, they account for less than 1% of all retroperitoneal tumors, making pelvic and perineal involvement exceptionally uncommon [6].

The location of these lesions in the ischioanal fossa and perineal region is exceptional and poses significant diagnostic and therapeutic challenges. Notably, masses in the ischioanal fossa are often misdiagnosed. This is due to the anatomical complexity of the deep pelvis and the difficulty in assessing its true extent via physical examination [7]. In adult patients, these lesions can easily mimic more frequent regional conditions such as perineal hernias, deep perianal abscesses, lipomas, or myxoid tumors, often leading to incomplete primary workups or inappropriate initial interventions [8]. Complete surgical excision is the preferred treatment option. However, in complex pelvic locations, surgery is associated with a high risk of incomplete resection and recurrence. This report details the effective management of a giant recurrent pelvic mass using a combined approach. This case has been reported in line with the SCARE 2023 guidelines [9].

## 2. Case Presentation

A 46-year-old female with no relevant clinical history presented with a seven-year history of a slowly growing mass in the right perineal region, causing pain and difficulty sitting. She had undergone two prior “surgical” or rather perineal drainages interventions at another hospital, the most recent two years earlier; no clinical records or operative reports from these procedures were available for review. She now presented with recurrence of a mass protruding through the right perineal and gluteal region, causing pain during defecation and while sitting.

**Figure 1:**
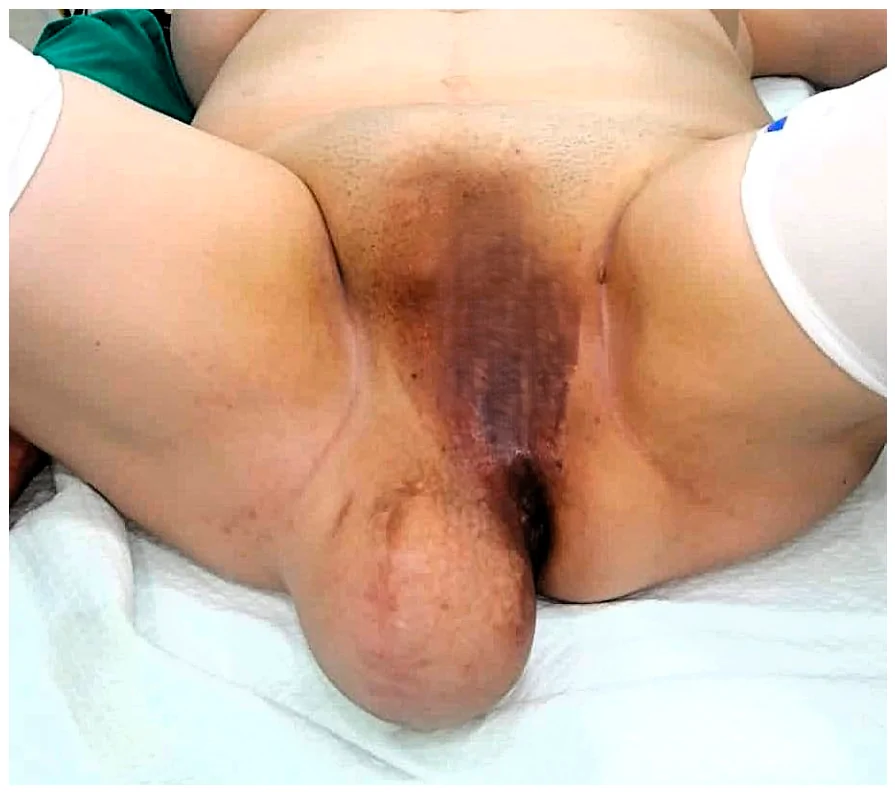
Clinical photographs show marked perineal asymmetry with a palpable mass in the right pararectal region, approximately 10 cm in diameter, and no signs of overlying skin infection.

Physical examination of the right inguinal region revealed a reducible right inguinal hernia. Inspection and palpation of the right pararectal region identified a mass approximately 10 cm in diameter, firm, mobile, and tender on manipulation (Figure 1). Digital rectal examination revealed a tonic anal sphincter with no palpable mass infiltration. Laboratory studies were unremarkable. Magnetic resonance imaging demonstrated a lesion of mixed consistency extending from the abdominal region to the right pararectal perineal region, without infiltration of adjacent structures (MRI imagens no available).

**Figure 2:**
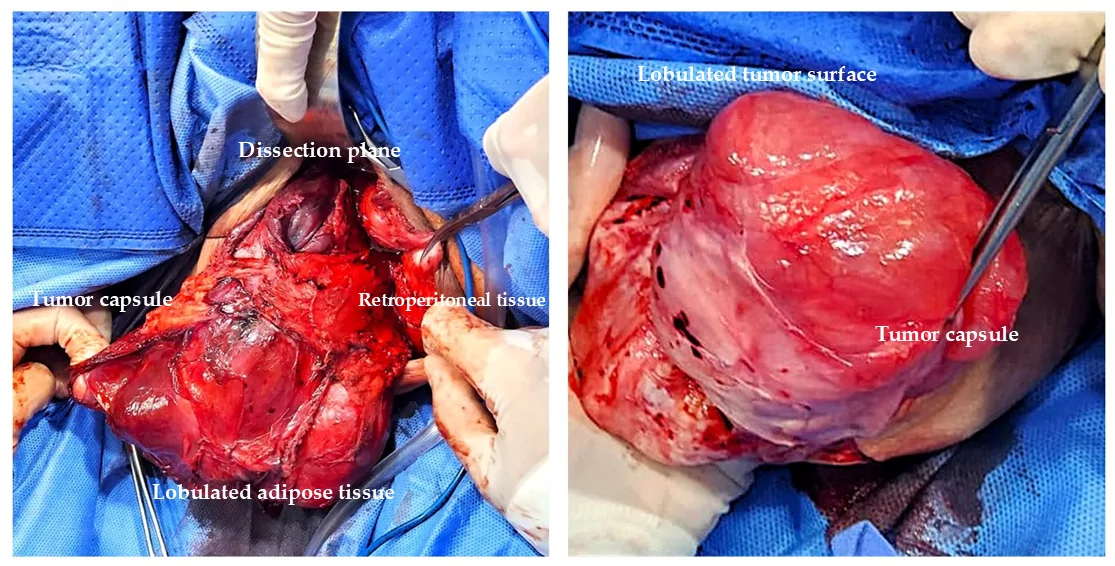
Descriptive intraoperative procedure and cystic mass identified in the right ischioanal fossa.

Considering the anatomical complexity and recurrent nature of the lesion, a planned surgical procedure was performed with the patient in dorsal decubitus and lithotomy position, using two simultaneous approaches: an exploratory laparotomy through a 15 cm supra- and infraumbilical midline incision, and a right pararectal perineal approach. No adhesion syndrome was observed within the abdominal cavity. A loculated cystic mass measuring 20 × 8 × 9.5 cm was identified in the right ischioanal fossa, in contact with the levator ani and obturator internus muscles, and projecting into the retroperitoneal pelvic cavity and the right presacral space; the mass was adherent to, but did not infiltrate, the musculoaponeurotic structures (Figure 2). The peritoneum was opened at the level of the rectosigmoid junction, and dissection was extended into the presacral space, where the right pararectal mass was identified and separated from the rectum at the pelvic level, continuing the dissection down to the perineal region.

**Figure 3:**
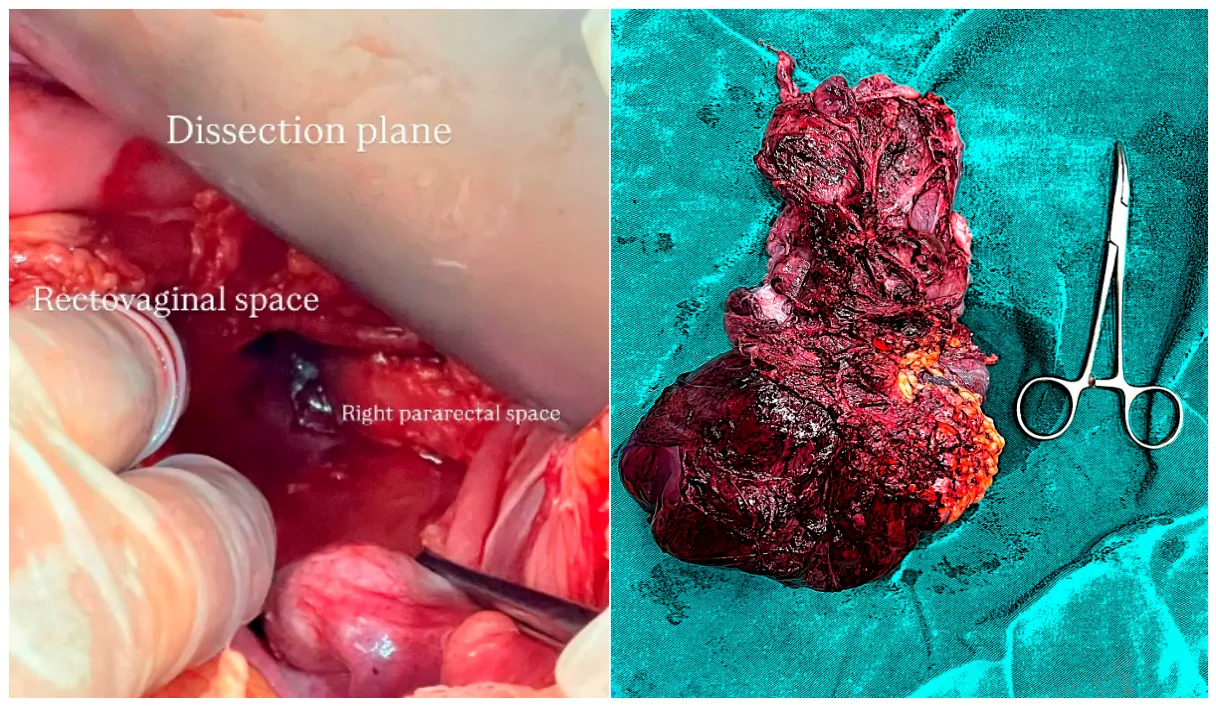
Gross specimen photograph of the surgically resected mass following complete en bloc excision, measuring 20 × 8 × 9.5 cm.

At this level, an incision was made and the mass was dissected with careful delineation and preservation of the external anal sphincter fibers, conserving the pelvic floor structures. Owing to the position of the mass, cranio-caudal communication between the abdominal and perineal cavities was created. The surgical specimen was removed completely en bloc, followed by reconstruction of the pelvic floor muscles and closure of the abdominal peritoneum. The surgical resection margins were negative, and a suction drain was placed before the patient was transferred to the recovery room (Figure 3).

**Figure 4:**
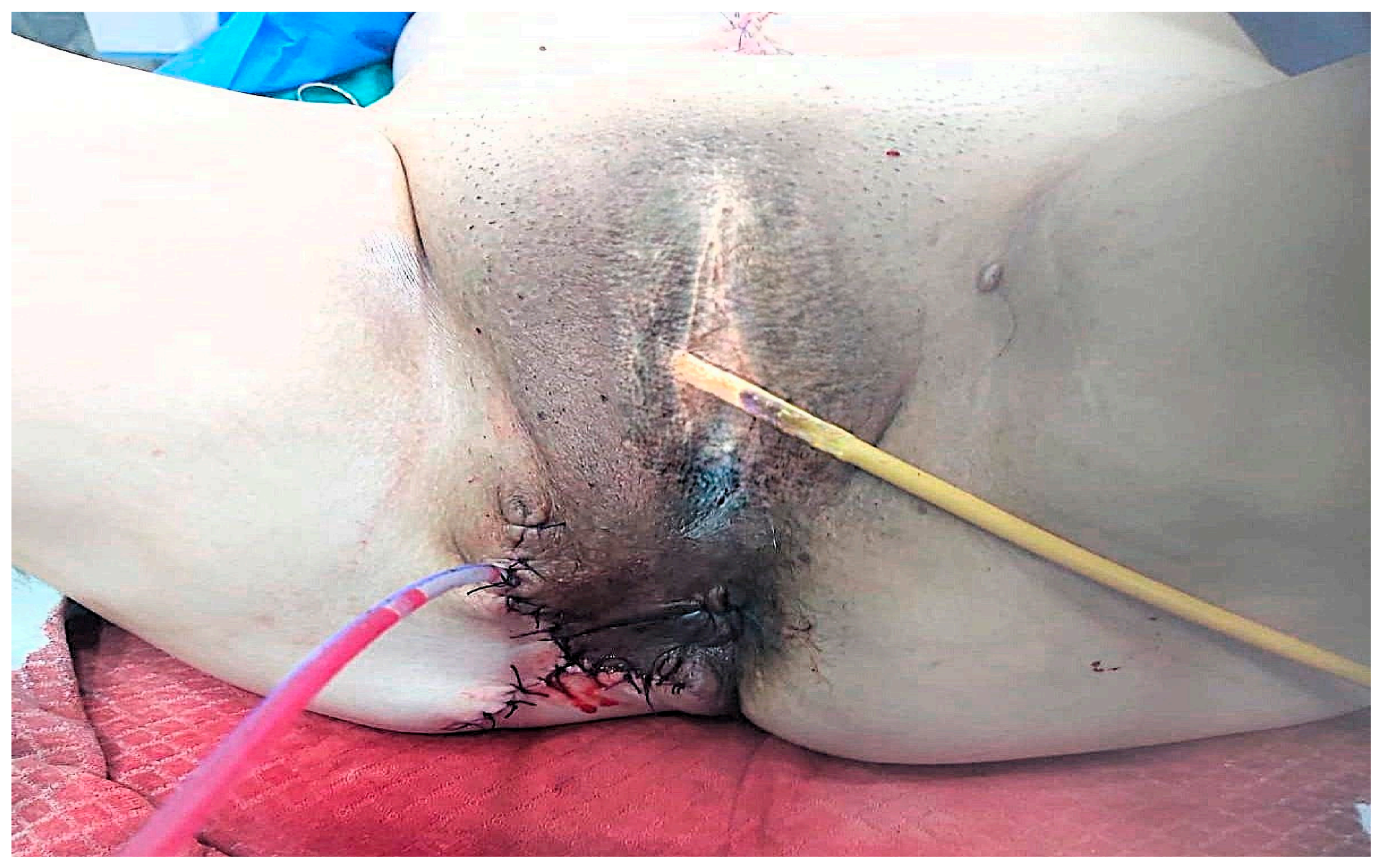
Postoperative photograph demonstrating the perineal surgical wound with preservation of anal sphincter integrity following the combined abdominal–perineal approach.

**Figure 5:**
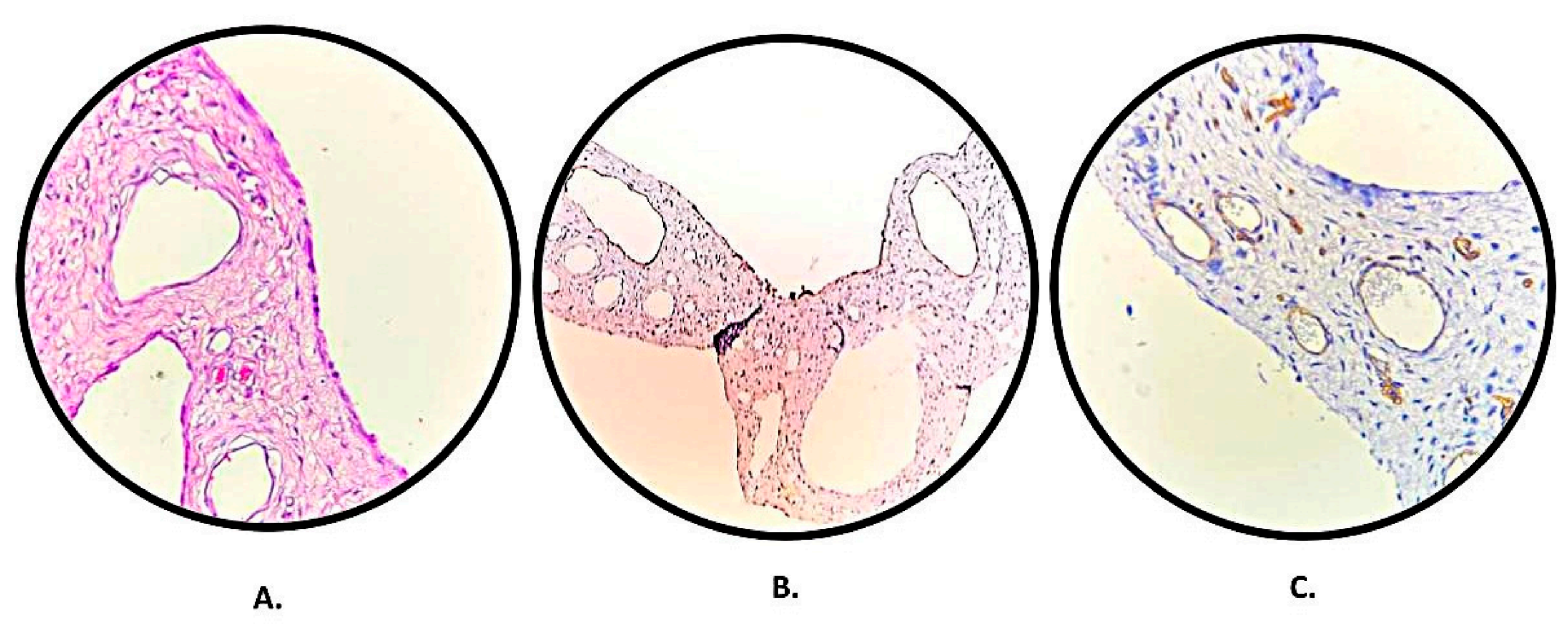
Photomicrographs of the resected specimen show dilated, thin-walled lymphatic channels lined by flattened endothelium, confirming the diagnosis of cystic lymphangioma. (**A**) Hematoxylin and eosin stain; (**B**) D2-40 immunostaining showing positive reactivity in macrocystic lymphatic channels; (**C**) CD34 immunostaining showing positive reactivity in small vessels. Original magnification ×40 for all panels.

The immediate postoperative course was favorable, with an early oral tolerance started at 8 h and adequate pain control. The patient had a bowel movement at 48 h without evidence of incontinence, and laboratory controls showed no leukocytosis or inflammatory response. The patient was discharged on postoperative day 5 without complications (Clavien–Dindo grade 0), and the drain was removed on day 8 without incident (Figure 4). The right inguinal hernia was repaired in a separate surgical stage using a Lichtenstein hernioplasty technique 3 months later at the same facility. The final histopathological examination with immunohistochemical analysis demonstrated positive staining for D2-40 (podoplanin) within the macrocystic lymphatic channels, confirming their lymphatic endothelial origin, while CD34 was positive in small adjacent vessels, consistent with a vascular component. These findings supported the diagnosis of lymphangioma (Figure 5). The patient was under follow-up; her last check-up six months ago post-surgery showed no recurrence.

## 3. Discussion

Adult pelvic lymphatic malformations are rare entities that pose significant diagnostic dilemmas. As described by Dessai et al., these lesions typically develop extrinsically within the presacral or ischiorectal space, thus limiting the utility of digital rectal examination (DRE) in accurately assessing their dimensions [10]. This finding is consistent with the observations made in our case study, in which a marked discrepancy was noted between clinical and surgical findings. Specifically, physical examination indicated the presence of a 10-centimetre mass, while intraoperative examination revealed a 20-centimetre lesion. This phenomenon, termed the “iceberg effect,” is a hallmark of tumors that extend into the deep pelvic retroperitoneum [11].

When comparing our case with other published reports of adult pelvic and retroperitoneal lymphangiomas, several distinct surgical and anatomical challenges emerge. For instance, Kim et al. described a giant pelvic lymphangioma in a 42-year-old female that extended into the perineum, mimicking a perineal mass [6]. While their case shared a similar trans-spatial extension into the lower perineum, it was successfully managed through a single transabdominal approach because it lacked prior surgical interventions or infiltrative fibrotic adhesions [6]. In contrast, our patient presented with a history of two previous surgical recurrences. Multiple recurrences significantly distort anatomical tissue planes, a complication well-documented by Sharma et al., who noted that recurrent retroperitoneal LMs tend to develop dense fibrous attachments to surrounding major pelvic vessels and pelvic floor muscles, dramatically increasing the technical difficulty of secondary operations [5].

The therapeutic strategy for these expansive lesions remains a subject of ongoing debate. Although some institutions have reported utilizing less invasive alternatives such as ultrasound-guided percutaneous sclerotherapy (bleomycin or doxycycline), these modalities demonstrate suboptimal efficacy in large, multiloculated, macrocystic lesions containing thick internal septations or substantial solid-fibrous components [12]. Consequently, the extant literature highlights the notion that complete surgical resection is the sole curative treatment, as conservative therapies are associated with high recurrence rates [13]. Gendvilaitė et al. observed that complex cystic variants tend to infiltrate tissue planes, rendering dissection arduous [13].

In this context, the simultaneous combined approach demonstrates a clear advantage over traditional single approaches. Whilst the isolated perineal approach offers minimal exposure (the probable cause of the patient’s previous recurrences), the combined approach allows for proximal vascular control and precise distal dissection, ensuring clear margins and sphincter preservation [7]. A similar combine-approach paradigm was successfully executed by Takahashi et al. for a recurrent retroperitoneal-pelvic mass, corroborating that a coordinated abdominoperineal entry provides the necessary visualization to safely detach the mass from the pelvic diaphragm while preserving sphincter integrity [8]. Notwithstanding the initial success, it is recognized that long-term follow-up is necessary in view of the chronic and recurrent nature of these pathologies.

Beyond the operative strategy, the diagnostic work-up of these lesions deserves particular attention, since preoperative imaging directly informs surgical planning in recurrent or infiltrative disease. Ultrasonography, computed tomography (CT), and magnetic resonance imaging (MRI) play complementary roles in characterizing pelvic and retroperitoneal lymphangiomas. In the largest available review of adult retroperitoneal lymphangioma, abdominal-pelvic CT was the most frequently used imaging modality, whereas MRI, performed in a minority of cases, has traditionally been regarded as the diagnostic test of choice given its superior soft-tissue contrast and multiplanar capability for delineating cystic septations, fluid content, and the relationship of the mass to adjacent pelvic floor structures [14]. More recently, Gerstner Saucedo et al. emphasized that CT and MRI should be viewed as complementary rather than competing studies: CT is useful for initial detection and characterization of a multiloculated, low-attenuation cystic mass, while MRI refines the differential diagnosis and better defines the extent of soft-tissue and vascular involvement [15].

Despite these advances, imaging remains inherently limited in reliably differentiating lymphangiomas from other cystic pelvic and retroperitoneal lesions, and definitive diagnosis continues to rely on histopathological and immunohistochemical confirmation after surgical resection, reinforcing the “iceberg phenomenon” already discussed in relation to physical examination in our patient [10,11]. Although the original MRI images were not retained for this report, the expected imaging features of pelvic and retroperitoneal lymphangiomas are well described in the literature. On CT, these lesions typically appear as thin-walled, multiloculated cystic masses with near-water attenuation and only mild septal or wall enhancement, without a solid component [16]. MRI characteristically shows low-to-intermediate signal on T1-weighted images and marked hyperintensity on T2-weighted images, with enhancement confined to the septa and cyst wall; any nodularity or solid enhancement should raise suspicion for malignancy, reinforcing histopathology as the diagnostic gold standard [15,16].

Strengths of this report include the detailed description of the combined surgical approach and the histopathological confirmation of the diagnosis. However, as a single case report, the findings are not generalizable; additionally, the MRi images are not available due to limited access, and the lack of prior operative records and the relatively short follow-up period constitute important limitations, given the risk of late recurrence described for these lesions.

## 4. Conclusions

Giant lymphangiomas with ischioanal extension require a high index of diagnostic suspicion because clinical evaluation frequently underestimates their true size. To ensure complete resection and limit damage to nearby pelvic structures, it is essential to plan surgery with the aid of advanced imaging and to apply a simultaneous combined abdominoperineal approach. This strategy aims to reduce the risk of recurrence in complex and recurrent cases.

## Data Availability

The data presented in this study are available on request from the corresponding author.

## Abbreviations

The following abbreviations are used in this manuscript:

LMs	Lymphatic malformations
ISSVA	International Society for the Study of Vascular Anomalies
DRE	Digital rectal examination
